# Supplementary material for: Urolithin A Protects Hepatocytes from Palmitic Acid-Induced ER Stress by Regulating Calcium Homeostasis in the MAM
Source: Biomolecules. 2024 Nov 26;14(12):1505. doi: 10.3390/biom14121505 (PMC11673756; doi:10.3390/biom14121505)
Supplement: Supplementary file 1 [file biomolecules-14-01505-s001.zip › biomolecules-3271382-supplementary.pdf]

Supplementary Materials

Urolithin A protects hepatocytes from palmitic acid-induced ER stress by regulating calcium homeostasis in the MAM

Gayoung Ryu[1], Minjeong Ko[1], Sooyeon Lee[1], Se In Park[1], Jin-Woong Choi[2,3], Joo Yeon Lee[2,3], Jin Young Kim[2,3] and Ho Jeong Kwon[1,*]

[1] Chemical Genomics Leader Research Laboratory, Department of Biotechnology, College of Life Science and Biotechnology, Yonsei University, Seoul 03722, Republic of Korea.

[2] Research Center of Bioconvergence Analysis, Korea Basic Science Institute, Ochang 28119, Republic of Korea.

[3] Critical Diseases Diagnostics Convergence Research Center, Korea Research Institute of Bioscience and Biotechnology, Daejeon 34141, Republic of Korea

* Correspondence: kwonhj@yonsei.ac.kr

Supplementary Figure S1.

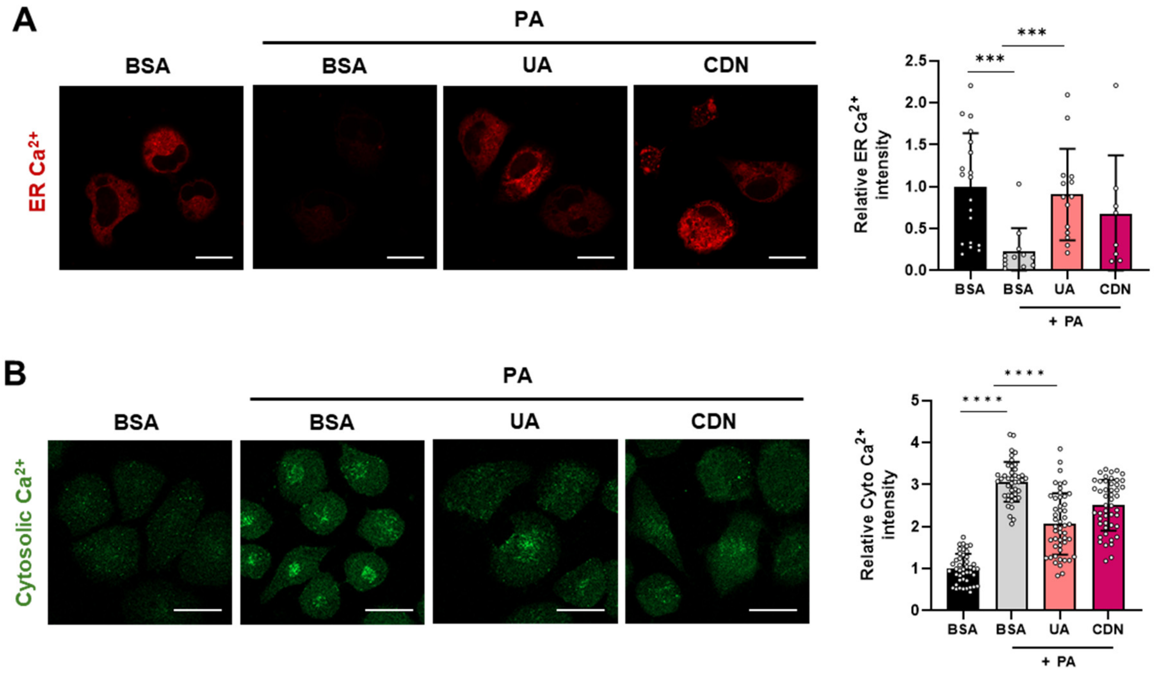

(**A**) ER calcium levels were measured in HepG2 cells treated with PA (500 μM) for 24 h, either alone or co-treated with UA (40 μM) and CDN1163 (10 μM) (scale bar: 10 μm). (**B**) Cytosolic calcium levels were assessed using the Fluo-4-AM after 24 h of treatment with PA, either alone or co-treated with UA (40 μM), and CDN1163 (10 μM) (scale bar: 20 μm). (***p < 0.001, ****p < 0.0001)

Supplementary Figure S2.

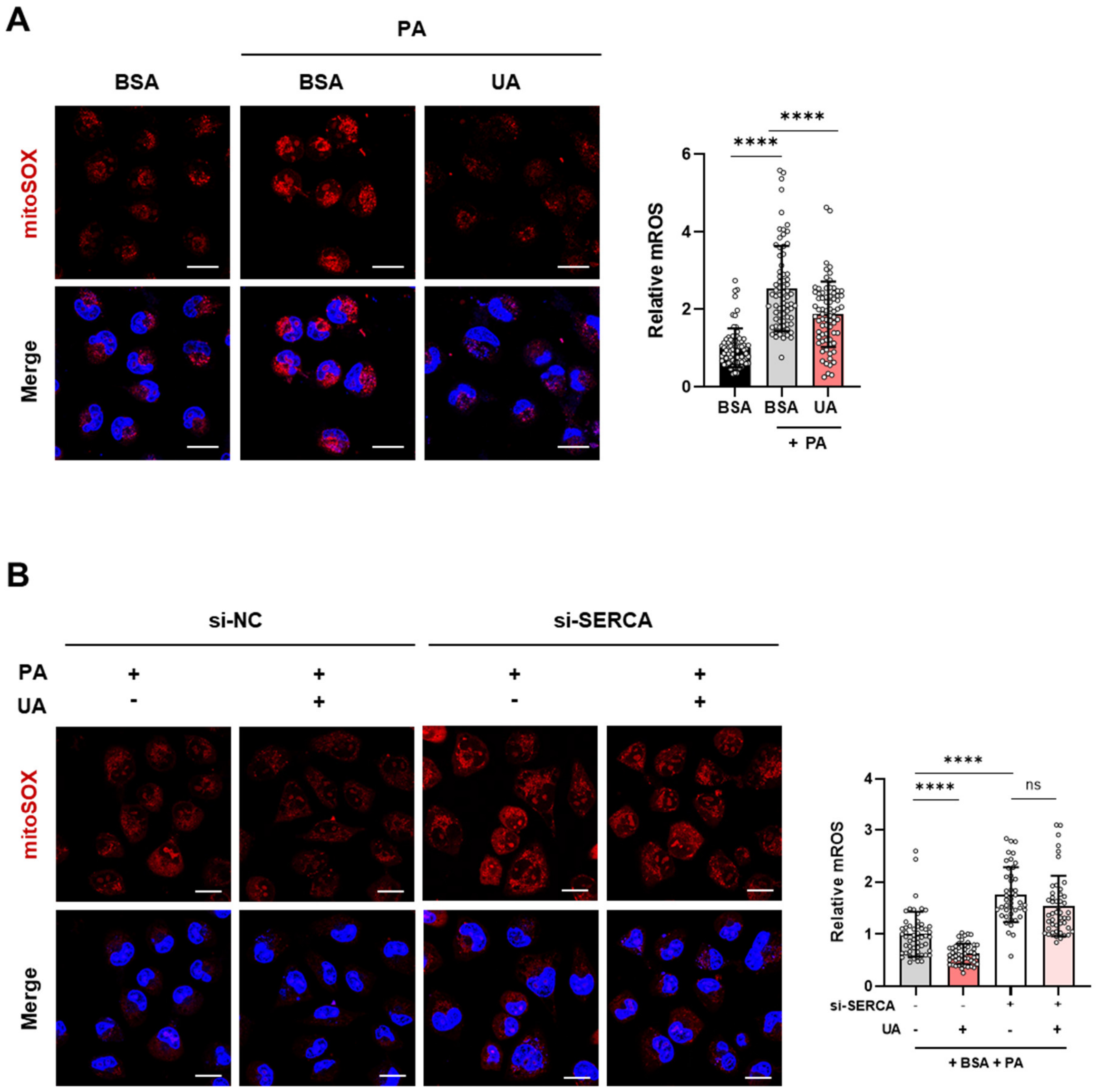

(**A**) HepG2 cells were treated with PA for 24 h with or without UA, followed by mitoSOX staining (scale bar: 20 μm). (**B**) Mitochondrial ROS levels were assessed to evaluate the effect of UA following SERCA knockdown in HepG2 cells (scale bar: 20 μm). (****$p < 0.0001$, ns: not significant)

Supplementary Figure S3.

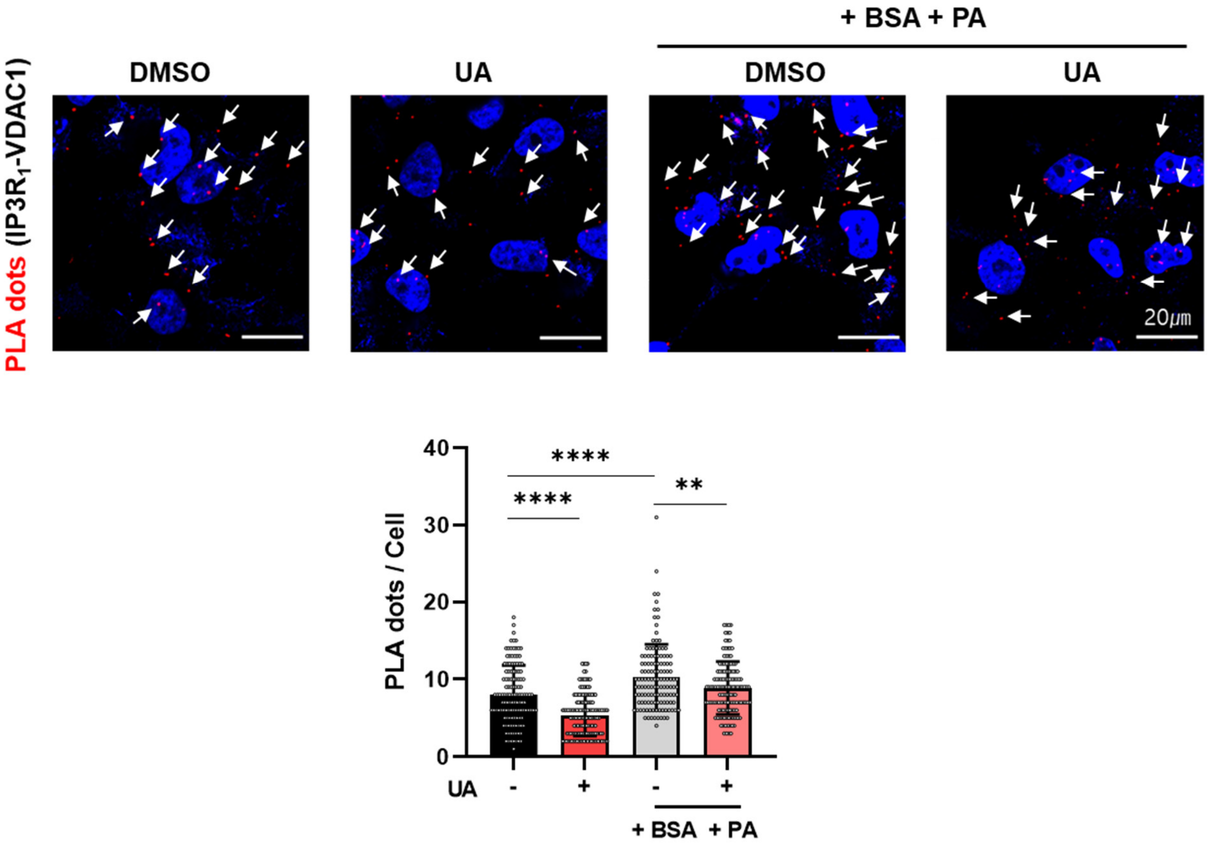

Proximity ligation assay (PLA) of IP3R and VDAC1 in HepG2 cells treated with PA (0.5 mM) and UA (40 μM) for 24 h was performed. The red fluorescent dots indicate sites of close proximity between the ER and mitochondria. Quantification was conducted by counting the number of red fluorescent dots (scale bar: 20 μm). (**$p < 0.01$, ****$p < 0.0001$)

Supplementary Figure S4

Uncropped Western blot images

A (Figure 3A)

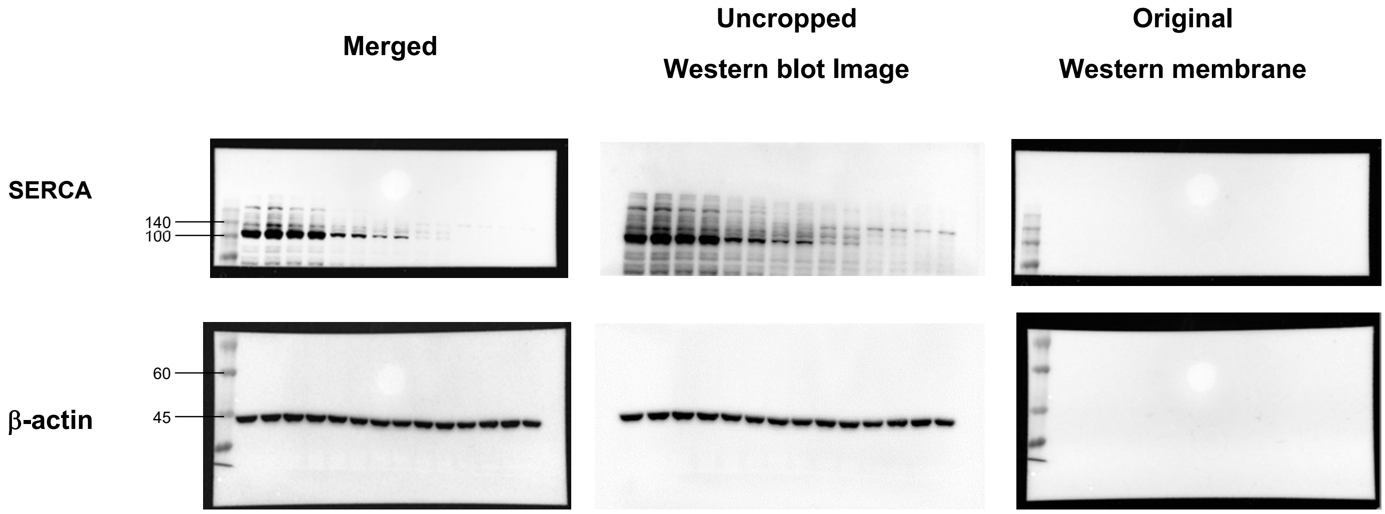

B (Figure 3B)

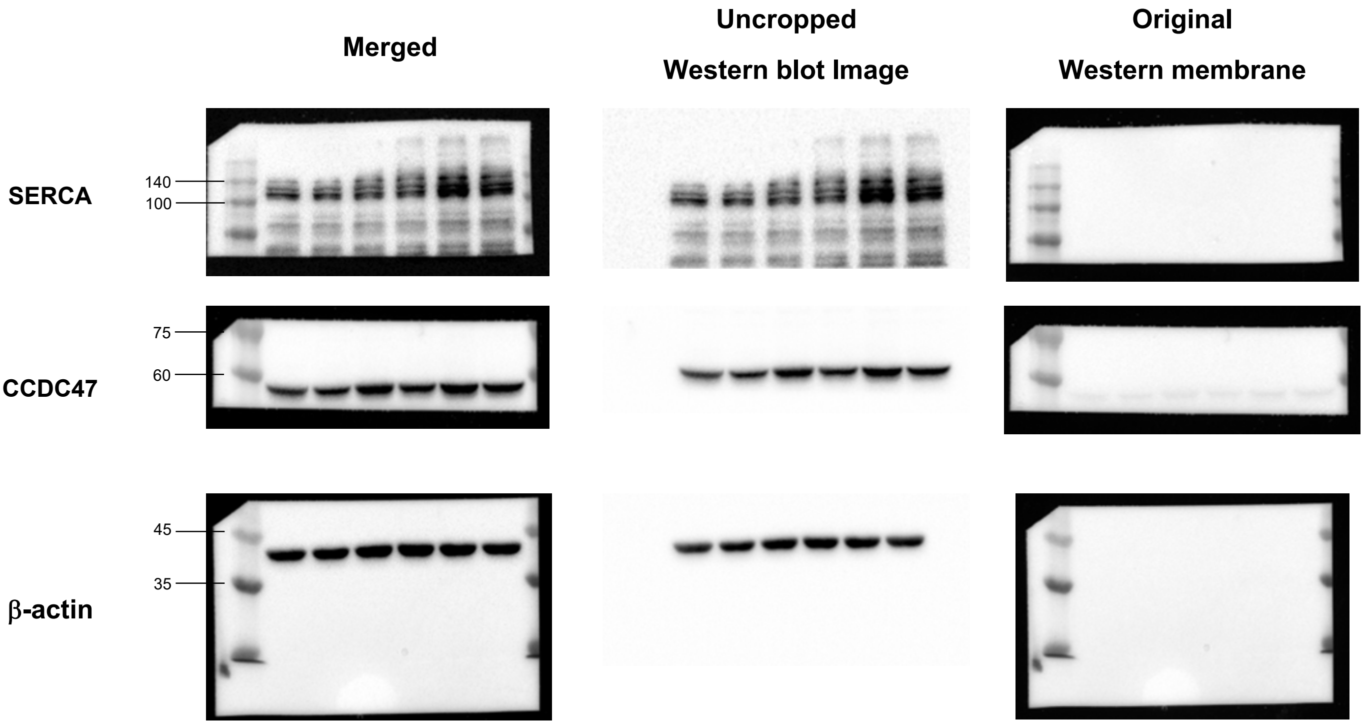

C (Figure 3F)

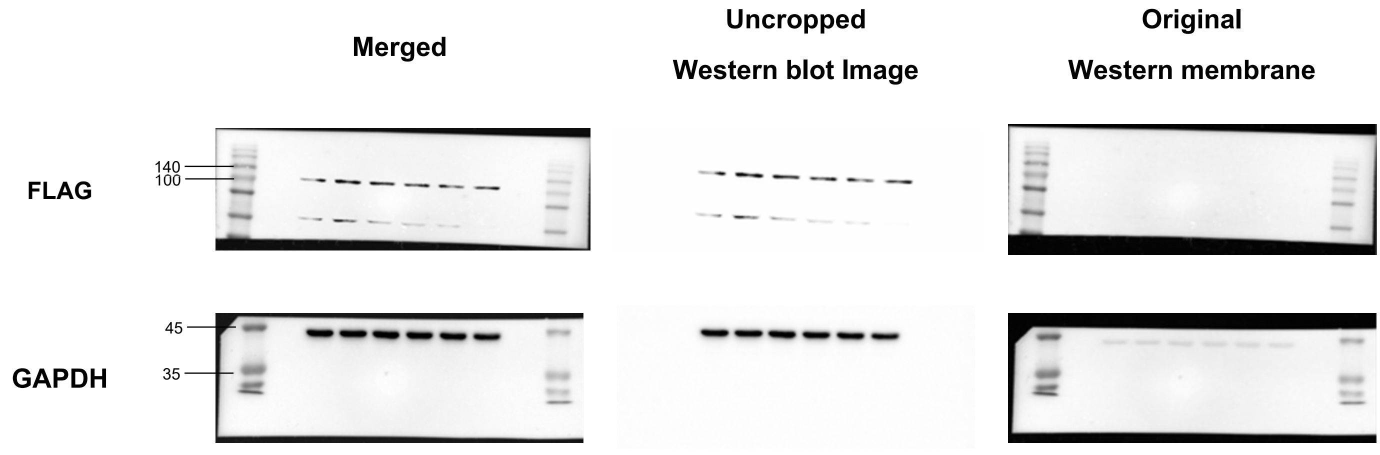

D (Figure 5A)

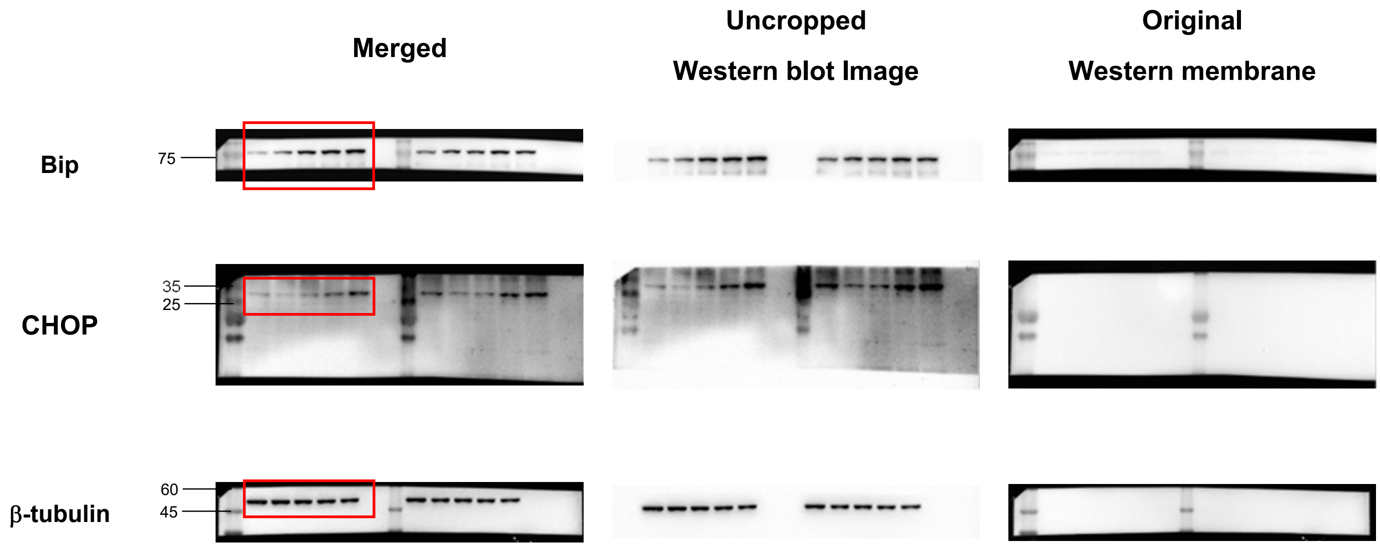

E (Figure 5B)

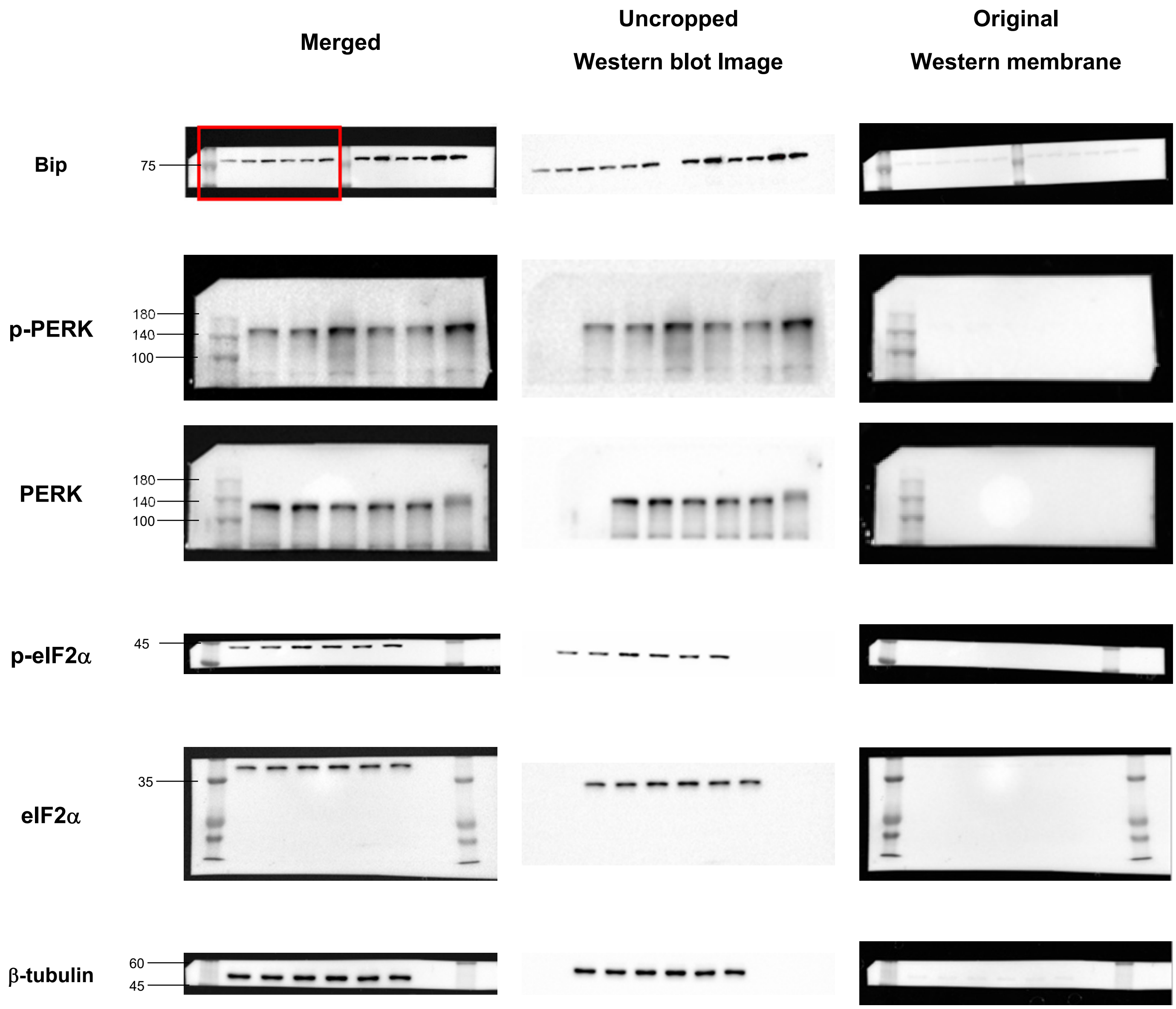

F (Figure 5C)

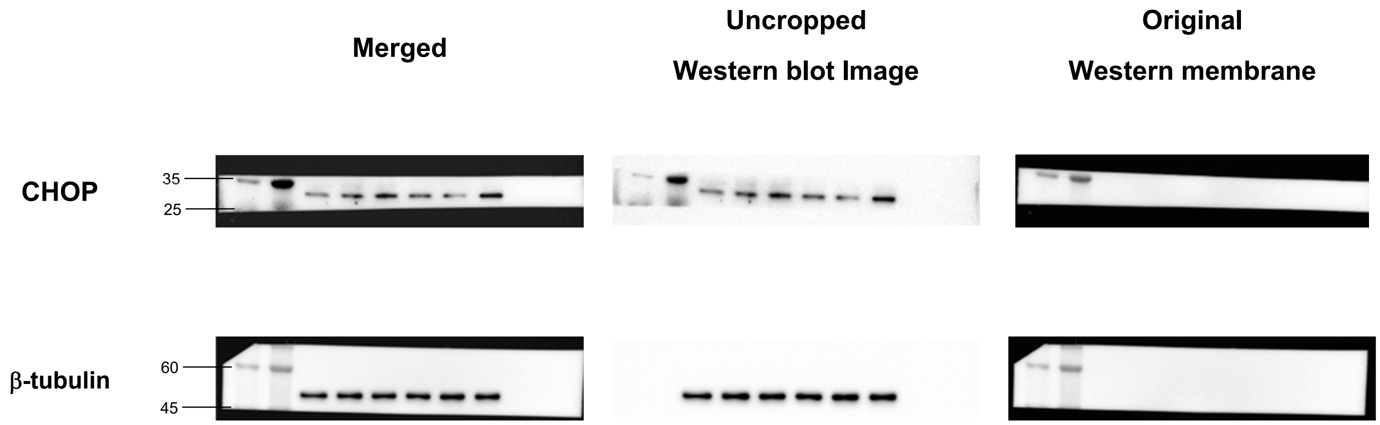

G (Figure 6D)

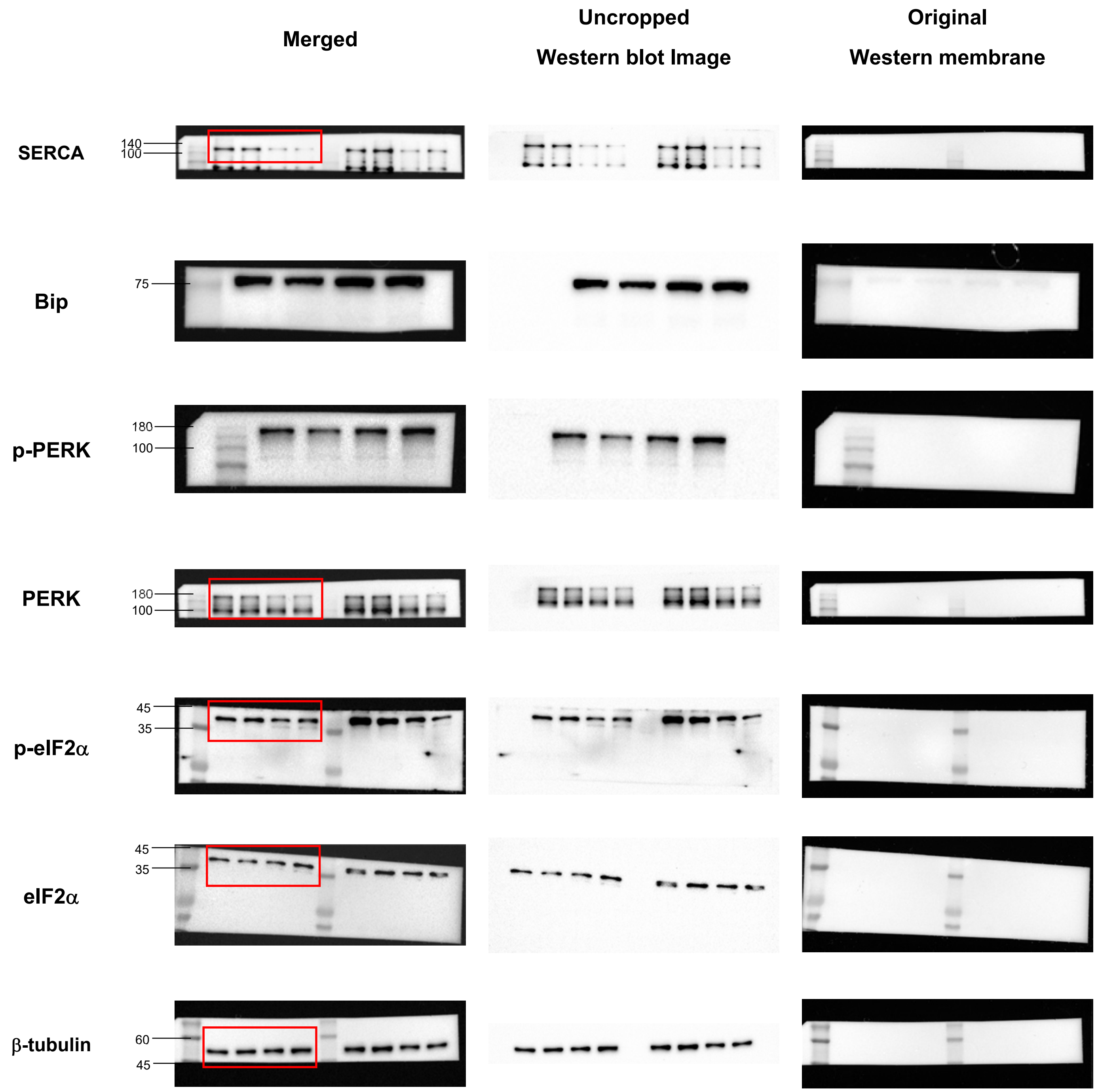
Merged
Uncropped
Western blot Image
Original
Western membrane
SERCA
140
100
Bip
75
p-PERK
180
100
PERK
180
100
p-eIF2α
45
35
eIF2α
45
35
β-tubulin
60
45
